# Physiological responses of young vegetative quinoa (*Chenopodium quinoa* Willd.) leaves to high temperatures under controlled conditions

**DOI:** 10.3389/fpls.2025.1737240

**Published:** 2026-01-12

**Authors:** Talya Fishbein, Ifat Matityahu, Daniel Bertero, Lior Rubinovich

**Affiliations:** 1 Northern Agriculture R&D, MIGAL – Galilee Research Institute, Kiryat Shmona, Israel; 2 Department of Biotechnology, Faculty of Science and Technology, Tel Hai College, Kiryat Shmona, Israel; 3 Department of Plant Production, Faculty of Agronomy and IFEVA-Conicet, University of Buenos Aires, Buenos Aires, Argentina

**Keywords:** carbon assimilation, *Chenopodium quinoa*, climate change, heat stress, protein content

## Abstract

Global warming is increasing the frequency of extreme heat events, posing major challenges for crop productivity and food security. Young vegetative quinoa (YVQ; *Chenopodium quinoa* Willd.) has emerged as a promising high-protein leafy crop, but little is known about its physiological performance under very high temperatures. This study examined the short-term responses of YVQ (cv. Peppermint) to a series of high-temperature gradients (30–55°C) under controlled conditions: 30-day-old plants were exposed to high temperatures for 5 days and evaluated before exposure, and 1 day (After 1d) and 14 days (After 14d) after exposure to assess their recovery. Despite exposure to peak temperatures of 55°C, no visible foliar injury was observed. Maximum quantum yield of photosystem II (Fv/Fm) remained stable across treatments, indicating protection of the photosynthetic apparatus. Leaf chlorophyll content index (CCI) increased at 40–49°C but plateaued at 55°C. In contrast, CO_2_ assimilation (A) and stomatal conductance (g_s_) declined sharply above 43°C but recovered at 43–49°C After 14d, suggesting transient impairment followed by acclimation. Exposure to 55°C resulted in a significant and non-recoverable reduction in gas-exchange parameters. Electrolyte leakage decreased at 43–46°C but increased markedly at 52–55°C, indicating a shift from stress priming to irreversible membrane injury. Total protein content reached its maximum following exposure to 55°C, likely reflecting accumulation of stress-induced proteins. Strong correlations were found between temperature and A, g_s_, electrolyte leakage, and CCI After 1d, but not After 14d. Temperature was also positively correlated to protein content After 14d. Overall, our findings suggest that temperatures of 43–49°C activated protective adaptation mechanisms, but temperatures ≥52°C exceeded compensatory capacity and caused irreversible impairment of carbon assimilation and membrane integrity. These findings demonstrate remarkable thermotolerance of YVQ and highlight its potential as a climate-resilient leafy crop for future hot environments.

## 1 Introduction

Quinoa (*Chenopodium quinoa* Willd., Amaranthaceae) is an underutilized protein-rich crop (Andreotti et al., 2022). Native to the Andean highlands and the lowlands of Central Chile in South America, this pseudo cereal is widely cultivated for its edible grains, primarily for human consumption (Bazile et al., 2016a, b). Quinoa is recognized for its resilience to climate stress and its capacity to thrive under harsh and unfavorable growing conditions (Choukr-Allah et al., 2016; Pulvento and Bazile, 2023). Quinoa grains possess a notably higher protein content than staple cereal crops such as barley, wheat, corn, and rice (Scanlin and Lewis, 2017; García-Parra et al., 2020). Its exceptional nutritional profile stems from a complete set of all nine essential amino acids (Pereira et al., 2019; Angeli et al., 2020). Moreover, it is gluten-free, making it suitable for individuals with celiac disease (Ceyhun Sezgin and Sanlier, 2019; Dakhili et al., 2019). Quinoa is therefore often marketed as a “functional food” or “superfood, “enhancing its global commercial appeal (Noulas et al., 2017). Consequently, quinoa cultivation has expanded significantly in recent years, with the crop now grown commercially in numerous regions beyond its native South America (Jacobsen, 2017; Alandia et al., 2020).

Young vegetative quinoa (YVQ), an unconventional leafy green vegetable, has recently gained scientific attention (Rubinovich et al., 2023, 2025; Huang et al., 2024). Resembling spinach in both texture and culinary versatility, the tender stalks and green leaves of the quinoa plant are highly nutritious and can be consumed either fresh or cooked (Adamczewska-Sowińska et al., 2021; Pathan and Siddiqui, 2022). YVQ can be cultivated year-round in open fields, greenhouses, or high tunnels (Pathan et al., 2023). Like quinoa grains, the leaves contain all nine essential amino acids; however, their protein content, expressed as a percentage of dry matter (DM), is significantly higher, ranging from 25% to 37%, compared to 9.1% to 15.7% in the grains (Pathan et al., 2019; Vazquez-Luna et al., 2019; Pathan and Siddiqui, 2022; Gómez et al., 2024). Furthermore, quinoa leaves have a higher protein content than other commonly consumed leafy greens such as spinach, chard, and broccoli (Vazquez-Luna et al., 2019). In addition to their superior protein profile, quinoa leaves are rich in copper, manganese, and potassium, and contain moderate levels of salt, calcium, phosphorus, and zinc (Pathan et al., 2019). Studies assessing the nutraceutical potential of YVQ have identified substantial levels of bioactive polyphenols in quinoa leaf extracts, which have been associated with inhibitory effects on the proliferation of prostate cancer cells (Gawlik-Dziki et al., 2013) and anti-inflammatory effects in macrophage cells (Khattib et al., 2025). Another notable advantage of YVQ is its lower concentration of saponins—bitter, antinutritive triterpenoid glycosides—compared to quinoa grains (Lim et al., 2020; Stoleru et al., 2022). YVQ is typically harvested between 30 and 62 days after sowing (DAS) (Pathan et al., 2023; Rubinovich et al., 2023), whereas detectable levels of sapogenins—the triterpenoid aglycone backbone of saponins—are only observed from 82 DAS onward (Dick Mastebroek et al., 2000; Abd El-Samad et al., 2018). Moreover, vegetative quinoa shoots at 60 DAS contain lower saponin levels than those at the reproductive stage (100 DAS) (Bernardo Solíz-Guerrero et al., 2002). These parameters, along with its high yield potential (Abd El-Samad et al., 2018; Adamczewska-Sowińska et al., 2021; Rubinovich et al., 2023, 2025), underscore the potential of cultivated YVQ as a novel, sustainable, and protein-rich leafy crop.

Climate change, associated with global warming, poses a significant threat to global food-production systems (Ramírez-Gil et al., 2019; Zandalinas et al., 2021). Projections indicate that climate change will lead to more frequent and intense heatwaves, with global mean annual temperatures expected to rise by up to 3°C by 2050 and 4.8°C by 2100 (Jagadish et al., 2020; Grüter et al., 2022). Elevated temperatures are perceived by plants as heat stress (Zandalinas et al., 2021), which can severely impair growth through physiological and developmental processes, ultimately leading to substantial yield losses (Fahad et al., 2017; Hassan et al., 2021). Heat stress disrupts key physiological processes in plants, including photosynthesis, respiration, stomatal conductance (g_s_), and the regulation of leaf water potential (Sharma and Manjeet, 2020; Shapira et al., 2021). Elevated temperatures can impair net carbon assimilation through multiple mechanisms, such as enhanced photorespiration and mitochondrial respiration, inactivation of Rubisco, and reduced activity of photosystem II (PSII) (Teskey et al., 2015; Slattery and Ort, 2019). Responses of g_s_ to heat stress are species- and context-dependent, with some plants showing increased conductance and others, a decline (Haworth et al., 2018; Eustis et al., 2020). At the cellular level, heat stress can cause direct damage, including increased thylakoid membrane fluidity, elevated production of reactive oxygen species (ROS), enzyme inactivation, loss of membrane integrity, impaired protein regulation, and protein degradation (Balfagón et al., 2019). These physiological and biochemical disruptions can lead to substantial tissue damage, often evident in twigs and leaves through visible symptoms such as sunburn, leaf senescence, inhibited growth, and discoloration. The severity of these effects depends on the intensity, duration, and developmental stage at which the heat stress occurs (Filewod and Thomas, 2014; Fahad et al., 2017).

Quinoa exhibits considerable variation in heat tolerance among cultivars, with differences reported in photosynthetic performance and reproductive parameters under heat stress. Physiological indices commonly used to evaluate quinoa’s heat tolerance include chlorophyll fluorescence, gas-exchange parameters such as CO_2_ assimilation and stomatal conductance, electrolyte leakage as a measure of membrane integrity, water use efficiency, and chlorophyll degradation (Eustis et al., 2020; Abbas et al., 2023). At the reproductive stage, pollen viability, grain and straw yield and composition are strong determinants of heat sensitivity (Hinojosa et al., 2019; Alvar-Beltrán et al., 2020; Tovar et al., 2020; Matías et al., 2021a, 2021b). While the effects of heat stress on quinoa grain and straw production have been previously studied (Hinojosa et al., 2019; Alvar-Beltrán et al., 2020; Tovar et al., 2020; Matías et al., 2021a, 2021b, 2022), the physiological responses of YVQ to high temperatures remain largely unknown. Given the increasing frequency of extreme heat events in recent years (Alon et al., 2022) and projected future extremes under climate-change scenarios approaching or exceeding 45-50 °C in quinoa-growing regions (Jagadish et al., 2020; Grüter et al., 2022), this study investigated the physiological responses and recovery capacity of YVQ plants following exposure to high and extreme temperatures of up to 55 °C under controlled conditions. While several studies have investigated the effects of such extreme temperatures in other plant species (Turhan et al., 2021; Aydogan and Turhan, 2022; Aydogan et al., 2025), to our knowledge, the present work represents the first investigation of YVQ under such conditions.

## 2 Materials and methods

### 2.1 Plant material

Quinoa seeds from accession Peppermint were obtained from Wild Garden Seed (Philomath, OR) and had an indicated germination rate of approximately 95%. This accession, with white panicles and white seeds, was selected for this study because it is a commercially available quinoa variety with high leaf biomass and quality, which performed well in previous summer experiments conducted in the experimental region (Rubinovich et al., 2025). The seeds were sown in 3-L pots containing a planting mixture of ‘Tuff Merom Golan’ (Alon Tavor, Israel) type ‘Ram 157’, a coconut-based substrate combined with peat soil and starter fertilizer. The pots were placed in a controlled-climate growth room (a walk-in growth room with internal dimensions of 4 m × 2.4 m × 2.35 m) located at the Northern Agriculture R&D research farm in northwest Israel (lat. 33°15’N, long. 35°62’E). Following germination and seedlings thinning, each pot contained three to five vigorous plants. The plants were grown for 30 days under controlled temperatures ranging from 20°C at night to 30°C during the day and exposed to artificial LED lighting from 0600 to 1600 h, with a light intensity of 850 µmol/m² s (ambient conditions). Plants were irrigated regularly via an automatic drip-irrigation system operating three times per day under ambient conditions and up to five times per day during high-temperature treatments. Each irrigation event applied water for 5 min at a rate of 2 L/h, ensuring non-limiting soil moisture conditions throughout the experiment. Therefore, given the ample irrigation, the physiological responses measured are primarily attributable to heat stress rather than water-related limitation or solute accumulation. Fertilization was carried out manually once a week using a 5-3–8 fertilizer (containing nitrate and ammonium nitrogen, phosphorus, and potassium), applied at a volume of 100 mL per plant, diluted 1:10 with water, starting from the second week after sowing.

### 2.2 Temperature-gradient treatments and temperature and relative humidity measurements

The temperature-gradient treatments were conducted on 30-day-old quinoa plants at the mature vegetative stage, just before inflorescence emergence. The plants were transferred to another controlled-climate growth room, where they were subjected to artificial heat stress by exposing them to six different high-temperature gradients for 5 days. Air temperature and relative humidity were continuously recorded at 10-min intervals by a miniature, waterproof Hobo data logger (MX2301A; Onset Corp., Bourne, MA). Temperatures of the different gradients (treatments) were designed to peak at 40°C, 43°C, 46°C, 49°C, 52°C, and 55°C. The selected temperature range was designed to encompass both moderate stress conditions and potential upper thermal thresholds. The temperature-gradient treatments were designed to simulate actual extreme heat events, similar to those observed in July 2020 at the research farm. In particular, the temperature gradients were designed to include diurnal fluctuations resembling the day-night patterns characteristic of Mediterranean summer heat events, as documented in a previous study (Shapira et al., 2021). Plants were also exposed to gradients which peaked at ~30°C to mimic ambient conditions (Control). Each temperature treatment was repeated three times, with five pots per repeat. Relative humidity was maintained at approximately 50–70% during daytime hours (**Supplementary Figure S1**) using a humidity-controlled ventilation system that activated an exhaust fan to regulate humidity levels within the controlled-climate growth room. Plants were subjected to light, irrigation, and fertilization as previously described. Following the different temperature treatments, plants were subjected to ambient conditions in the first growth room.

### 2.3 Leaf measurements

**Figure 1:**
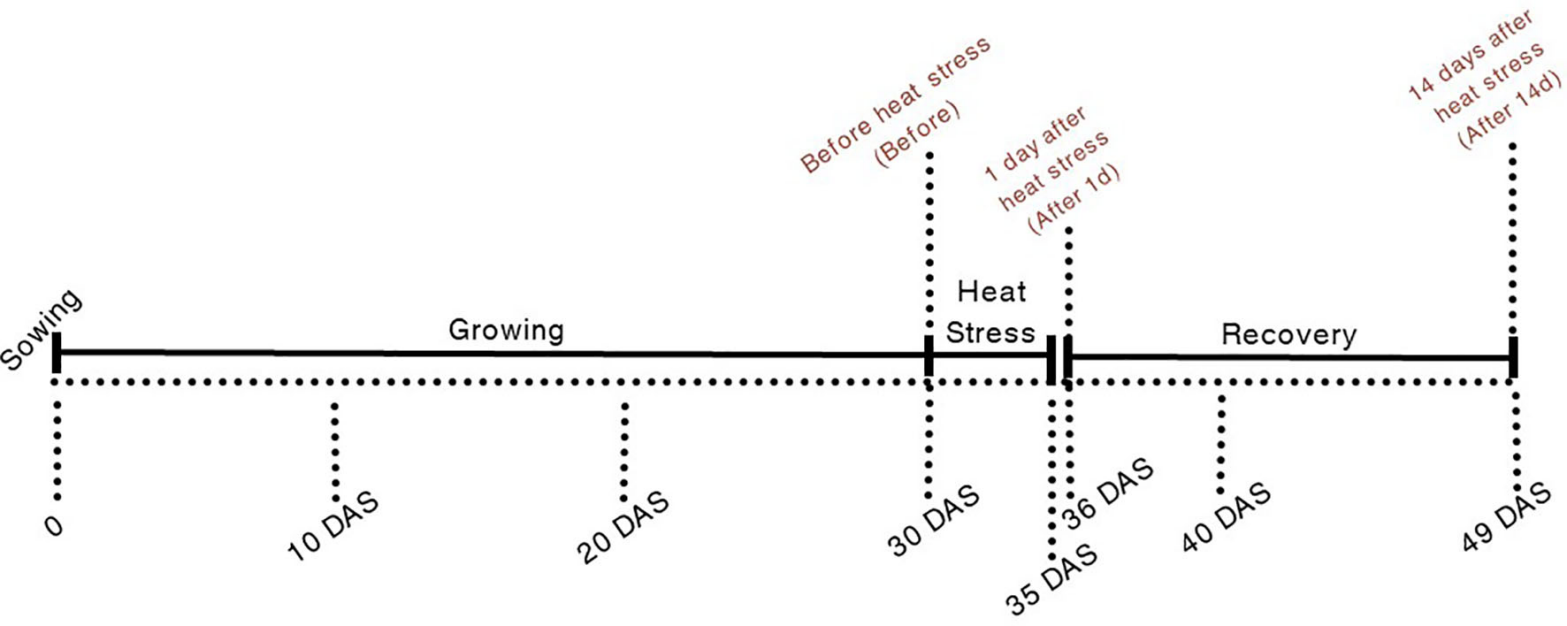
Experimental timeline of high-temperature treatment in quinoa. Quinoa plants were sown and grown under ambient conditions until 30 DAS. At this stage, plants were subjected to a 5-day heat-stress period (30–35 DAS), followed by a recovery phase that extended to 49 DAS. Key time points for leaf physiological measurements included: before heat stress (30 DAS, “Before”), 1 day after heat stress (36 DAS, “After 1d”), and 14 days after heat stress (49 DAS, “After 14d”).

Leaf measurements were taken at three different time points (**Figure 1**): (i) 30 DAS, before exposure to the temperature gradients (Before); (ii) 36 DAS, 1 day after the end of exposure to the temperature gradients (After 1d) and (iii) 49 DAS, 14 days after exposure to the temperature gradients (After 14d), to assess plant recovery. The selected timing was designed to allow plants to stabilize following the high temperature treatments and to capture both the early physiological response and the potential recovery capacity following exposure to high temperatures. Each pot served as one biological replicate (n = 15 per treatment). For leaf-damage assessment, chlorophyll *a* fluorescence, chlorophyll concentration, and leaf-level light-intensity and gas-exchange measurements, data were obtained from at least three fully expanded leaves per plant, all located at the same position relative to the shoot tip (technical replicates). These technical replicates were averaged to obtain a single independent biological replicate value used in the statistical analyses.

#### 2.3.1 Leaf-damage assessment

Leaf damage was assessed by a blind test in which two surveyors independently scored each leaf on a scale of 0–5, with 0 representing no apparent leaf damage and 5, maximum leaf damage, as shown in previous studies (Weil et al., 2019; Shapira et al., 2021; Lahak et al., 2024). To document visible phenotypic effects of the temperature treatments, potted plants were photographed using a digital camera at the three time points of the experiment (Before, After 1d, and After 14d).

#### 2.3.2 Chlorophyll *a* fluorescence analysis and chlorophyll measurements

A FluorPen FP100 portable fluorometer (Photon Systems Instruments, Drasov, Czech Republic) was used to measure the fluorescence of chlorophyll *a* in total darkness (at least 20 min of dark adaptation), and to calculate the maximum quantum yield of PSII (Fv/Fm) (Schreiber et al., 1986; Kramer et al., 2004; Baker, 2008). A chlorophyll meter (Apogee MC-100, Apogee Instruments, Logan, UT) was used to measure the leaf chlorophyll concentration index (CCI).

#### 2.3.3 Leaf-level light intensity and gas-exchange measurements

CO_2_-assimilation rates (A) and transpiration rates were measured using a LI-6800 portable photosynthesis system (clear-top 6-cm^2^ chamber with a mounted small light source, LI-COR, Lincoln, NE). The light source was set to a light intensity of 1000 µmol/m² s. Airflow into the leaf chamber was maintained at approximately 700 μmol/s, with CO_2_ concentration set at 415 ppm, and boundary-layer conductance to water vapor at approximately 3 mol/m^2^ s. The chamber temperature and relative humidity were set to ambient. Mature attached leaves were measured in the growth room at midday, under standardised ambient conditions, regardless of the plants’ prior high-temperature treatment. Thus, gas-exchange parameters reflect the physiological status of leaves before exposure to high temperatures or during recovery, rather than instantaneous responses to the high-temperature gradients. Selected leaves were positioned four to five leaves back from the branch tip. While the leaves were in the chamber, care was taken to keep them oriented toward the light source. The LI-COR device calculated g_s_ (stomatal conductance to water vapor).

#### 2.3.4 Electrolyte-leakage measurements

Three fully expanded quinoa leaves were collected from plants in each pot. In this case, each pot served as one biological replicate (n = 15 per treatment). Measurements were derived from pooled leaf samples from at least three plants per pot to ensure representative biological variability. Leaves were gently rinsed with distilled water to remove surface ions. Two leaf discs (1.1 cm diameter) were excised from each leaf using a cork borer, yielding a total of six discs per biological repeat. The discs were placed in test tubes containing 6 mL deionized water and incubated at room temperature for 16 h. Initial electrolyte leakage (EC1) was determined by measuring the electrical conductivity of the bathing solution with an HI2003–02 edge conductivity meter (Hanna Instruments, Woonsocket, RI). To obtain total electrolyte leakage, the same tubes were subsequently heated at 90°C for 2 h, cooled to room temperature, and conductivity was measured again (EC2) (Cueva-Flores et al., 2024). Electrolyte leakage was expressed as percentage of the total conductivity according to the formula:

electrolyte leakage (%) = EC1/EC2.

### 2.4 Plant protein-content determination

Plant samples of each treatment were collected at the After 14d time point and dried at 65°C for 48 h. The Kjeldahl method (AOAC, 2019) was used to determine the nitrogen content of each quinoa sample at the Milouda and Migal laboratories (Kiryat Shmona, Israel), where protein content (% of DM) was also estimated. The conversion factor used to transform nitrogen into protein was 6.25 (AOAC, 2019). Each pot served as one biological replicate (n = 15 per treatment). Measurements were derived from pooled samples of at least three plants per pot to ensure representative biological variability.

### 2.5 Statistical analysis

Data were analyzed using repeated-measures ANOVA (linear mixed model) in JMP version 18.2.2 (SAS Institute, Cary, NC) to account for temporal dependencies in the dataset and to test for interactions using ‘temperature’, ‘time point’ (DAS) and their interaction as fixed effects, ‘time point’ as a repeated factor, and ‘replicate’ as a random effect. When significant effects were detected, pairwise comparisons among time points within each treatment were performed by Tukey–HSD test. Results from After 1d and After 14d were also independently subjected to a two-tailed Pearson correlation matrix using the ‘corrplot’ package in RStudio (Boston, MA), in the programming language R.

## 3 Results

### 3.1 Actual temperature gradients in the controlled-climate growth room

**Figure 2:**
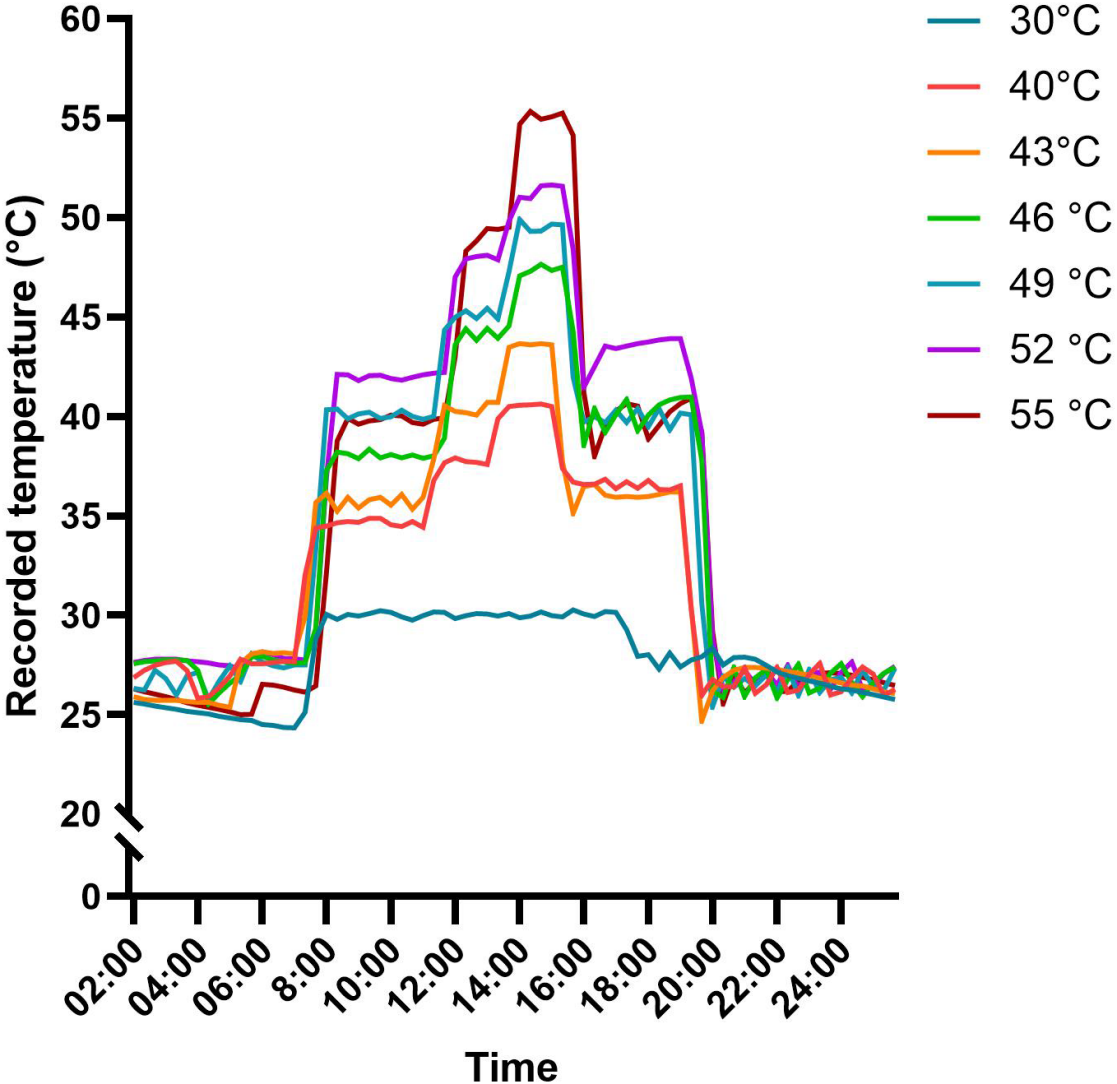
Recorded temperatures for the different gradients. Quinoa plants were exposed to different stepwise temperature gradients in a controlled-climate growth room. Graph shows representative 24-h temperature profiles recorded for each gradient treatment.

The controlled-climate growth room created stepped temperature gradients. **Figure 2** shows a representative 24-h temperature profile for each treatment. The recorded peak temperatures closely matched the predesigned 30°C, 40°C, 43°C, 46°C, 49°C, 52°C, and 55°C treatments, reaching actual maxima of 30.2°C, 40.6°C, 43.7°C, 47.7°C, 49.9°C, 51.6°C, and 55.3°C, respectively (**Figure 2**).

### 3.2 Effect of high temperatures on leaf damage

**Figure 3:**
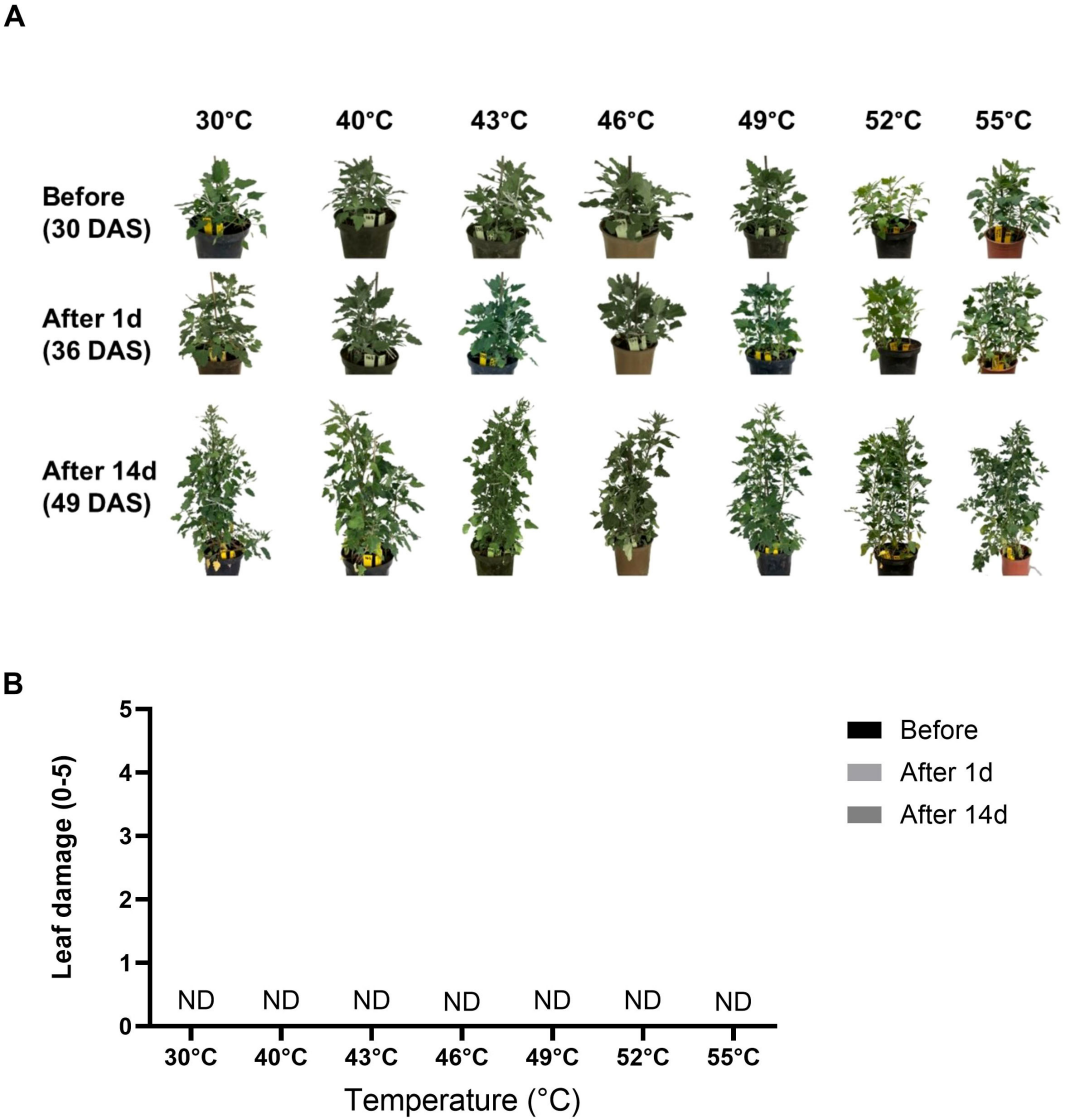
Leaf-damage assessment. **(A)** Representative photos of potted quinoa plants subjected to the different high-temperature treatments. **(B)** Leaf damage was assessed and scored on a scale of 0–5, with 0 representing no apparent damage and 5 representing maximum damage. ND – not detected. Photos were taken and leaf damage was assessed at three time points: before exposure (Before, 30 DAS), 1 day after exposure (After 1d, 36 DAS), and 14 days after exposure (After 14d, 49 DAS) to the temperature gradients. Values are means ± SE of 15 replicates (n = 15), each comprised of at least three different leaves per plant.

**Figure 3A** shows representative photos of potted quinoa plants subjected to the different temperature treatments. Before the temperature treatments, there was no visible damage to any of the quinoa plants. This was also the case in the After 1d and After 14d control plants. Interestingly, leaf-damage assessment showed no visible damage for any of the high-temperature treatments at either After 1d or After 14d time points (**Figure 3B**).

### 3.3 Effect of high temperatures on Fv/Fm and chlorophyll content

**Figure 4:**
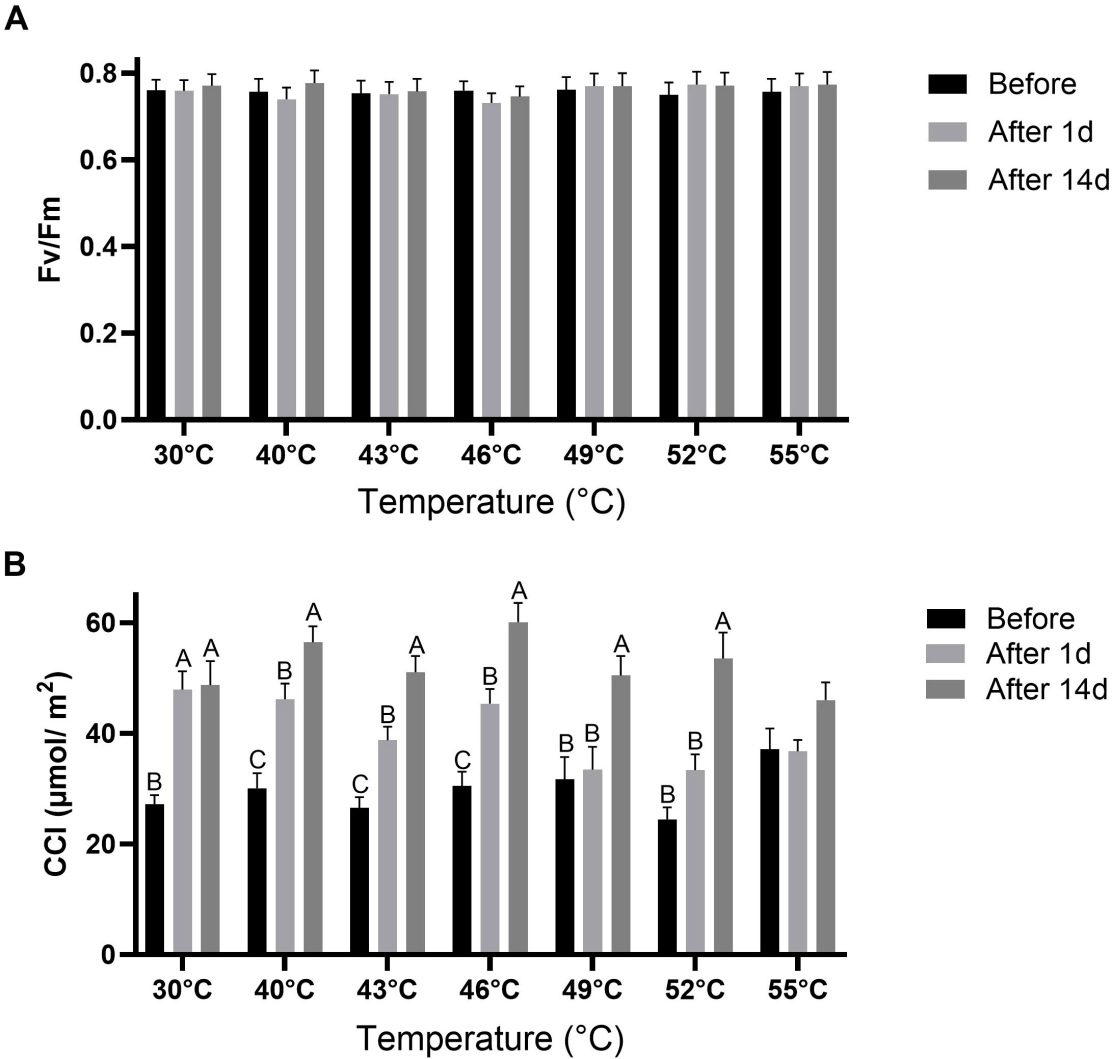
Maximum quantum yield of PSII (Fv/Fm) and leaf chlorophyll content (CCI) in leaves of quinoa plants under control and high-temperature treatments. **(A)** Chlorophyll *a* fluorescence was recorded after dark adaptation to calculate Fv/Fm. **(B)** CCI. Parameters were measured at three time points: before exposure (Before), 1 day after exposure (After 1d), and 14 days after exposure (After 14d) to the temperature gradients. Values are means ± SE of 15 replicates (n = 15), each comprised of at least three different leaves per plant. Different letters for a given temperature treatment indicate significant difference (Tukey-HSD, *P* < 0.05).

Before the temperature treatments, Fv/Fm values were similar across treatments, ranging from 0.75 to 0.76 (**Figure 4A**). Fv/Fm was not significantly (*P* > 0.05) affected After 1d or After 14d in any of the temperature treatments. Before the temperature treatments, CCI values ranged from 27 to 37 µmol/m^2^ across treatments (**Figure 4B**). In the control treatment, CCI increased significantly (*P* < 0.05) After 1d, and remained at that level After 14d. In the 40°C, 43°C, and 46°C treatments, CCI increased significantly (*P* < 0.05) After 1d and was increased further (*P* < 0.05) After 14d. In the 49°C and 52 °C treatments, CCI did not change significantly (*P* > 0.05) After 1d, but increased significantly (*P* < 0.05) After 14d. There was no significant (*P* > 0.05) difference between CCI at the different measurement time points for the 55°C treatment.

### 3.4 Effect of high temperatures on gas-exchange parameters

**Figure 5:**
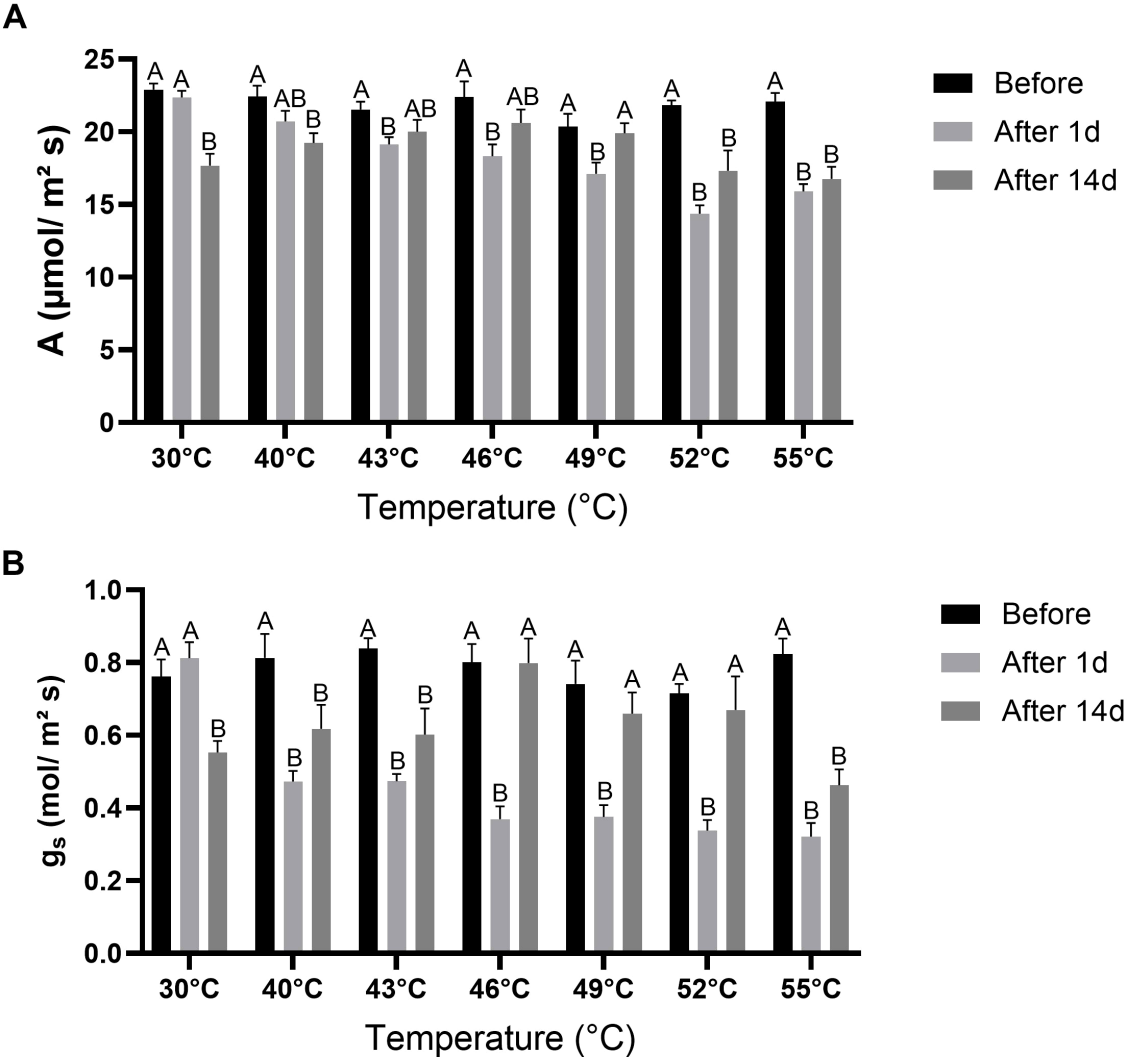
Gas-exchange parameters in quinoa plants under control and high-temperature treatments. CO_2_ assimilation **(A)** and stomatal conductance (g_s_) were measured and calculated by the LI-COR system. **(A)** A values. **(B)** g_s_ values. Parameters were measured at three time points: before exposure (Before), 1 day after exposure (After 1d), and 14 days after exposure (After 14d) to the temperature gradients. Values are means ± SE of 15 replicates (n = 15), each comprised of at least three different leaves per plant. Different letters for a given temperature treatment indicate significant difference (Tukey-HSD, *P* < 0.05).

Before the temperature treatments, rates of CO_2_ assimilation (A) were relatively similar across treatments, ranging between 20.4 and 22.9 μmol/m² s (**Figure 5A**). In the control treatment, A remained similar After 1d, but was significantly (*P* < 0.05) reduced After 14d. In all high-temperature treatments except 40°C, A was significantly (*P* < 0.05) reduced After 1d, with the greatest reductions observed at 52°C and 55°C. After 14d, partial recovery was evident in the 43°C, 46°C, and 49°C treatments, where A values did not differ significantly (*P* > 0.05) from Before values. In contrast, in the 40°C, 52°C, and 55°C treatments, A remained significantly (*P* < 0.05) lower than Before values. Before the temperature treatments, g_s_ values were consistent across treatments, ranging from 0.72 to 0.84 mol/m² s (**Figure 5B**). In the control treatment, g_s_ was not significantly (*P* > 0.05) affected After 1d but declined significantly (*P* < 0.05) After 14d. In all high-temperature treatments, g_s_ decreased significantly (*P* < 0.05) After 1d. After 14d, g_s_ remained significantly (*P* < 0.05) lower than Before values in the 40°C, 43°C, and 55°C treatments, but recovered in the 46°C, 49°C, and 52°C treatments.

### 3.5 Effect of high temperatures on electrolyte leakage

**Figure 6:**
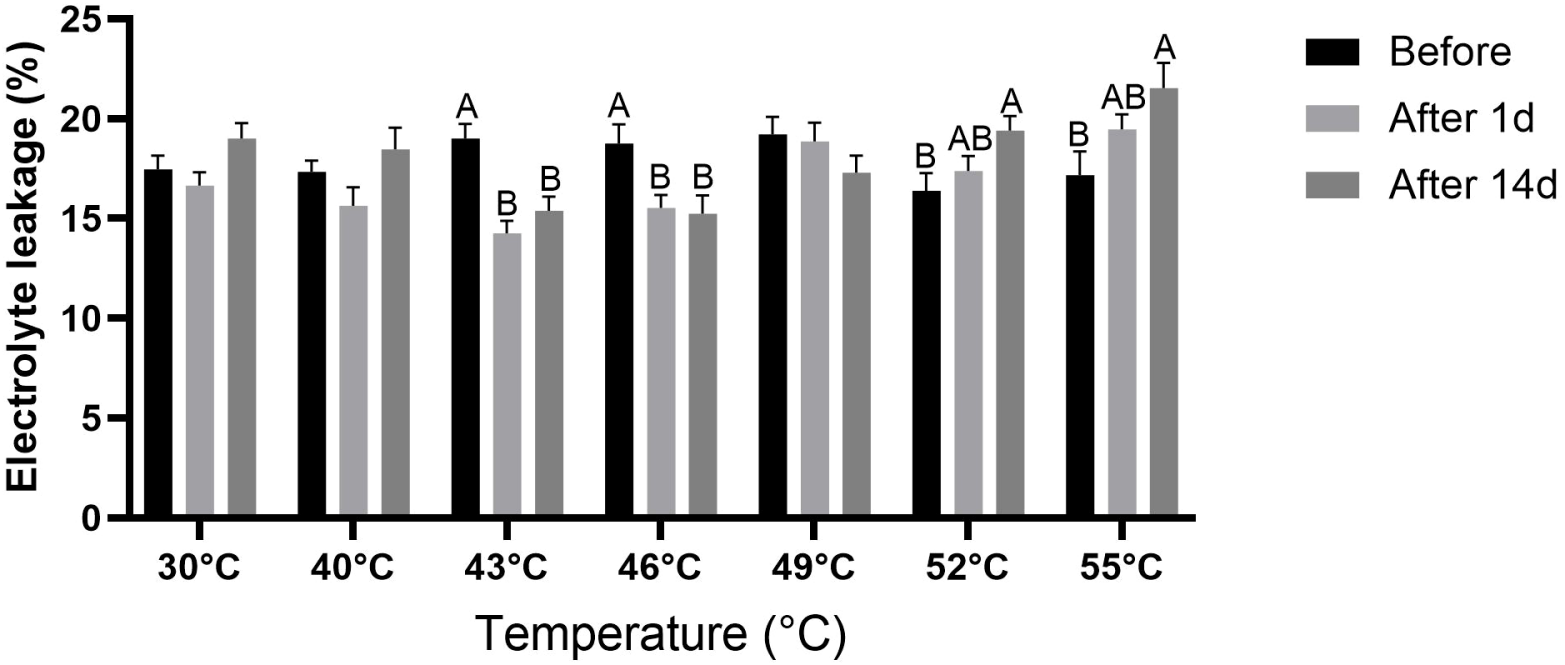
Electrolyte leakage in leaves of quinoa plants under control and high-temperature treatments. Measurements were taken at three time points: before exposure (Before), 1 day after exposure (After 1d), and 14 days after exposure (After 14d) to the temperature gradients. Values are means ± SE of 15 replicates (n = 15), each comprised of at least three different leaves per plant. Different letters for a given temperature treatment indicate significant difference (Tukey-HSD, *P* < 0.05).

Before the temperature treatments, electrolyte-leakage values were similar across treatments, ranging from 16.4% to 19.2% (**Figure 6**). In the control treatment, electrolyte leakage was not significantly (*P* > 0.05) affected After 1d or After 14d. This was also the case for the 40°C and 49°C treatments. In both 43°C and 46°C treatments, electrolyte leakage was significantly (*P* < 0.05) reduced After 1d and After 14d. In both 52°C and 55°C treatments, electrolyte leakage was slightly elevated After 1d, and significantly (*P* < 0.05) elevated After 14d compared to the initial measurement time point.

### 3.6 Effect of high temperatures on leaf protein content

**Figure 7:**
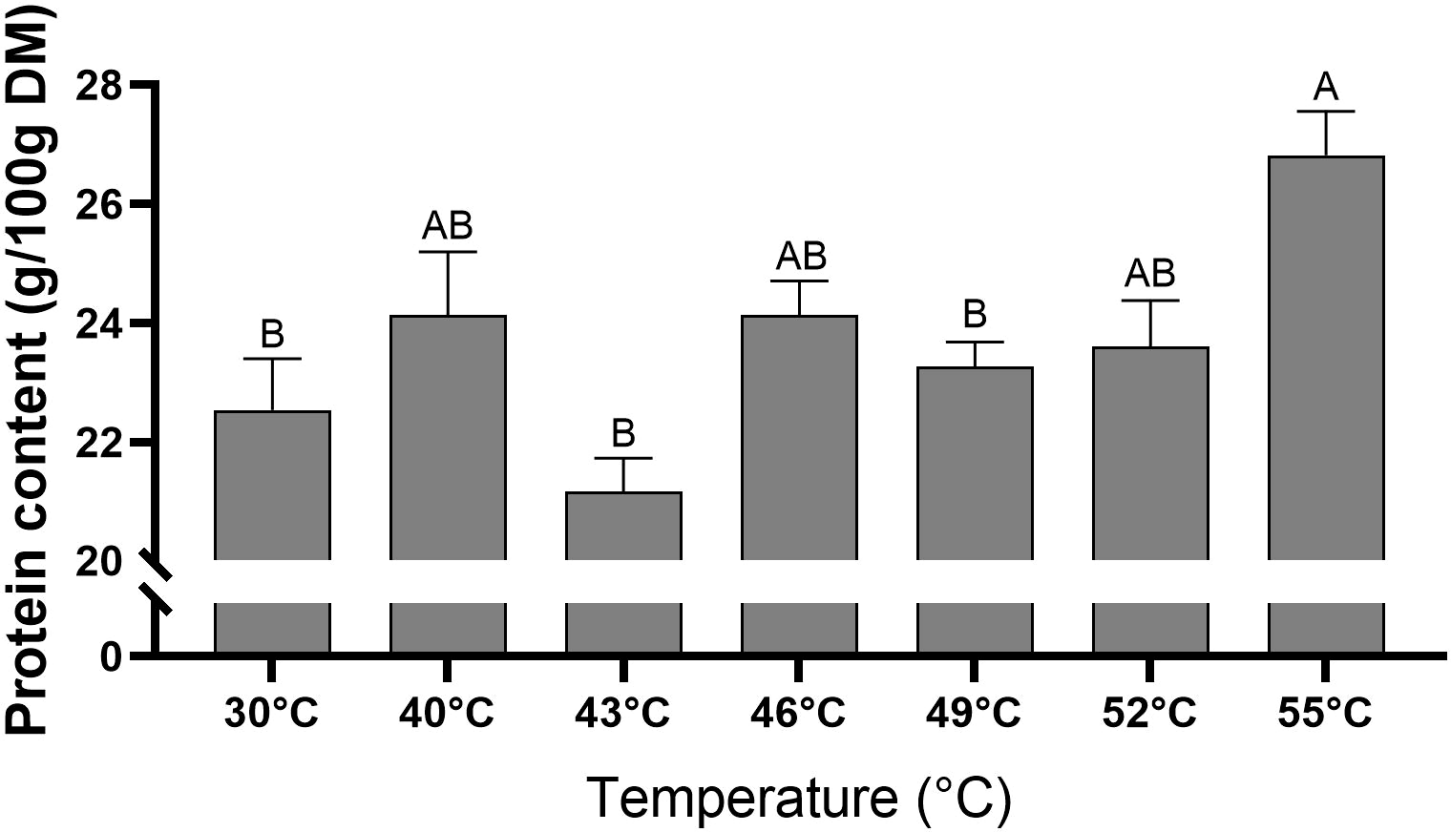
Protein content in quinoa plants under control and high-temperature treatments. Plant samples of each treatment were collected 14 days after exposure to the temperature gradients (After 14d). Values are means ± SE of 15 replicates (n = 15), each comprised of at least three plants. Different letters for a given temperature treatment indicate significant difference (Tukey-HSD, *P* < 0.05).

Leaf protein content ranged between 21.2 and 26.8 g/100 g DM (**Figure 7**). The lowest protein contents were recorded in the 30°C, 43°C, and 49°C treatments, which were significantly (*P* < 0.05) lower than the highest value observed at 55°C. The 40°C, 46°C, and 52°C treatments exhibited intermediate protein content levels that were not significantly (*P* > 0.05) different from those of the other temperature treatments.

### 3.7 Correlation between measured temperature and physiological parameters

**Figure 8:**
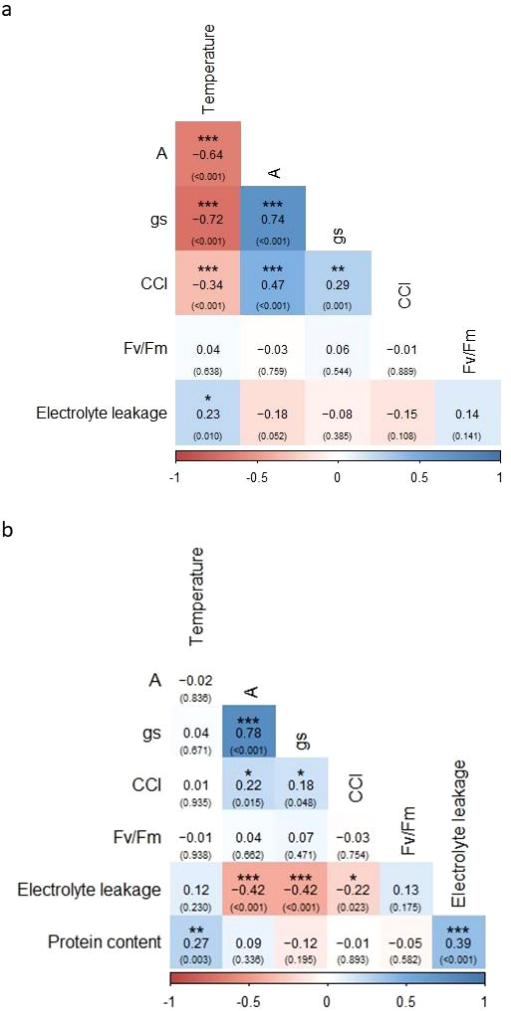
Pearson correlation coefficients among temperature, CO_2_ assimilation **(A)**, stomatal conductance to water vapor (g_s_), leaf chlorophyll content index (CCI), maximum quantum yield of PSII (Fv/Fm), electrolyte leakage and protein content for **(A)** After 1d and **(B)** After 14d samples. Colors reflect correlation values between every two variables. Blue and red colors represent positive and negative correlations, respectively. *P*-values are shown in parentheses, and asterisks indicate significance of Pearson correlation values (*P < 0.05, **P < 0.01, ***P < 0.001) within each box.

Of the 15 correlation coefficients evaluated After 1d, 7 exhibited significant values (*P* < 0.05; **Figure 8A**). Temperature was negatively correlated with A, g_s_, and CCI, but positively correlated with electrolyte leakage. Its correlation with Fv/Fm was not significant (*P* > 0.05); A was strongly and positively correlated with g_s_ and moderately correlated with CCI, whereas its correlations with Fv/Fm and electrolyte leakage were not significant (*P* > 0.05); g_s_ was positively correlated with CCI, but its correlations with Fv/Fm and electrolyte leakage were not significant (*P* > 0.05). The correlations of CCI with Fv/Fm and electrolyte leakage were not significant (*P* > 0.05), and Fv/Fm showed no significant correlations with the other variables (*P* > 0.05) (**Figure 8A**).

Of the 21 correlation coefficients evaluated After 14d, 8 exhibited significant values (*P* < 0.05; **Figure 8B**). Temperature was only positively correlated with protein content; A remained strongly and positively correlated with g_s_ and moderately correlated with CCI, but was negatively correlated with electrolyte leakage, while its correlations with Fv/Fm and protein content were not significant (*P* > 0.05); g_s_ showed similar patterns, being positively correlated with CCI and negatively correlated with electrolyte leakage, with no significant correlations to Fv/Fm or protein content (*P* > 0.05). CCI was negatively correlated with electrolyte leakage, and was not significantly correlated to Fv/Fm or protein content (*P* > 0.05), and Fv/Fm showed no significant correlations with the other variables (*P* > 0.05). Electrolyte leakage was positively correlated with protein content (**Figure 8B**).

The linear mixed model revealed significant main effects of temperature on several parameters (**Table 1**). A, g_s_, CCI, electrolyte leakage, and protein content were all significantly affected by temperature (*P* < 0.01 to *P* < 0.001), whereas Fv/Fm showed no significant (*P* > 0.05) response. The measuring time point (DAS) also had a significant effect on A, g_s_, and CCI, whereas that on Fv/Fm and electrolyte leakage remained non-significant (*P* > 0.05). Treatment-by-time point interactions showed significant effects for A, g_s_, CCI, and electrolyte leakage (*P* < 0.01 to *P* < 0.001), whereas Fv/Fm showed no significant response (*P* > 0.05). Protein content was not evaluated across time points as it was measured only at the end of the experiment (After 14d).

**Table 1:** Summary of fixed-effect tests from linear mixed model analyses of temperature, time point (DAS), and their interaction on physiological parameters in the experimental plants.

Effect	A	g_s_	Fv/Fm	CCI	Electrolyte leakage	Protein content
Temperature	***	**	ns	**	***	***
Time point (DAS)	***	***	ns	***	ns	NA
Temperature * Time point (DAS)	***	***	ns	**	***	NA

A, CO_2_ assimilation; g_s_, stomatal conductance to water vapor; Fv/Fm, maximum quantum yield of PSII; CCI, leaf chlorophyll content. Significance at *P < 0.05, **P < 0.01, ***P < 0.001; ns, non-significant; NA, not applicable.

## 4 Discussion

This study offers comprehensive insights into the physiological responses of YVQ leaves to various high-temperature gradients under controlled conditions. Our findings reveal a remarkable degree of resilience in YVQ plants, which maintained intact leaf morphology with no visible damage, even after exposure to several days of peak extreme temperatures of up to 55°C (**Figure 2**). Despite this apparent tolerance, closer examination of photosynthetic performance, chlorophyll content, membrane stability, and protein content indicated that YVQ undergoes distinct adjustments in its response to high temperatures, some of which may underlie its ability to sustain these harsh conditions.

Chlorophyll *a* fluorescence is a reliable and precise method for detecting damage to PSII and is widely regarded as one of the most effective approaches for studying heat tolerance in plants *in vivo* (Sharma et al., 2015). A reduction in Fv/Fm values relative to non-stressed conditions generally reflects impaired electron transport in the photosynthetic apparatus, potentially leading to photoinhibition (Baker, 2008). For example, Fv/Fm values decreased significantly in cotton plants exposed to 40°C compared to 30°C, and in water spinach exposed to 42°C compared to 25°C, indicating damage to the plants’ photosynthetic apparatus (Guo et al., 2020; van der Westhuizen et al., 2020). Young heat-sensitive tomato plants also exhibited lower Fv/Fm values compared to heat-tolerant genotypes following exposure to high temperatures under controlled conditions (Poudyal et al., 2019). An interesting outcome of the current study was the stability of the Fv/Fm values in the quinoa leaves, which remained unaffected across all treatments (**Figure 4**). Moreover, there was no significant (*P* > 0.05) correlation between temperature and Fv/Fm (**Figure 8**), and the linear mixed model showed that it was not significantly (*P* > 0.05) affected by temperature (**Table 1**). Thus, the high temperatures did not induce any damage leading to chronic photoinhibition in ‘Peppermint’ quinoa leaves, which would otherwise manifest as a sustained reduction in Fv/Fm values (Maxwell and Johnson, 2000; Shapira et al., 2021). These findings align with a previous study showing that heat-tolerant quinoa accessions maintained stable Fv/Fm values when young potted plants were exposed to high temperatures of 45°C (Eustis et al., 2020).

The increase in CCI in the leaves of Control quinoa plants at the After 1d time point (**Figure 4**) likely reflects a natural developmental process (Eustis et al., 2020). A similar trend was observed in plants exposed to the 40–46°C treatments; interestingly, in these cases, CCI continued to increase during the recovery phase (After 14d), similar to findings in ‘Kaslaea’ and ‘17GR’ quinoa accessions following 45°C heat treatment (Eustis et al., 2020). These results contrast with the expected chlorophyll degradation under stress due to lipid peroxidation in membranes and chloroplasts (Fahad et al., 2017; Hassan et al., 2021), suggesting that quinoa leaves may upregulate chlorophyll synthesis or reduce its degradation under these high temperatures. The increase in CCI was delayed under the higher 49°C and 52°C treatments compared to the other treatments. The absence of a CCI increase under the 55°C treatment indicates a threshold beyond which chlorophyll synthesis may be partially impaired, supporting the view that exposure to 55°C can override protective responses.

Gas-exchange measurements revealed a more sensitive response to high temperatures (**Figure 5**). Both A and g_s_ were significantly affected by temperature, time point and their interaction (**Table 1**), consistent with the central role of photosynthetic gas exchange in plant responses to heat stress. In the Control treatment, gas-exchange parameters were not affected After 1d. In contrast, these parameters were negatively correlated with temperature at this time point (**Figure 8A**). After 1d, A and g_s_ declined significantly after exposure to the high temperatures of 43°C and >40°C, respectively, with the lowest values observed at 52– 55°C. A reduction in photosynthetic rate and g_s_ was also found in water spinach plants exposed to 35°C, 40°C, or 45°C, and wucai (*Brassica campestris*) plants exposed to 40°C (Zou et al., 2017; Wang et al., 2023). Interestingly, After 14d, both A and g_s_ were significantly reduced in the Control plants, possibly due to a natural developmental process. However, at this time point, neither parameter was correlated with temperature (**Figure 8B**). Moreover, A and g_s_ recovered to their Before values in the 43–49°C treatments and the 46–52°C treatments, respectively. This indicates that following exposure to these high temperatures, YVQ plants retain the capacity to restore photosynthetic activity once the heat subsides. A previous study reported similar results, showing elevated photosynthetic rate and g_s_ in several quinoa accessions following exposure to heat treatments of 40°C or 45°C (Hinojosa et al., 2019; Eustis et al., 2020). However, in those studies, the effects of higher temperatures were not investigated. The lack of recovery for both A (at 52–55°C) and g_s_ (at 55°C) suggests that these temperatures are the threshold beyond which photosynthesis becomes irreversibly impaired, reinforcing the notion that extreme heat can override protective mechanisms, as seen with the CCI measurements (**Figure 4**). The strong positive correlation between A and g_s_ observed here (**Figure 8**) underscores the central role of stomatal regulation in carbon assimilation under heat stress (Slattery and Ort, 2019).

At the cellular level, electrolyte leakage is a reliable method for assessing cell membrane integrity and is commonly used as an indicator of direct heat-induced injury (Demidchik et al., 2014). Indeed, this parameter was not affected in the control treatment (**Figure 6**). Electrolyte leakage showed a biphasic response to high temperature: following exposure to 43–46°C, there was an unexpected reduction in electrolyte leakage, whereas at 52°C and 55°C, leakage increased, particularly After 14d. Reduced electrolyte leakage at intermediate temperatures may reflect stress priming, whereby moderate heat induces protective adjustments that stabilize the membranes (Bäurle, 2016; Hassan et al., 2021). In contrast, the elevated electrolyte leakage at the extreme temperatures of 52°C and 55°C is consistent with heat-induced cellular damage (Gulen and Eris, 2004; Hassan et al., 2021). The significant correlations observed between electrolyte leakage, carbon assimilation, g_s_, CCI, and protein content After 14d (**Figure 8B**) suggest that membrane stability is closely integrated with broader cellular responses to stress in quinoa leaves following the recovery period.

Although heat stress can upregulate specific proteins—such as heat-shock proteins, ROS-detoxifying enzymes, and other stress-related proteins—overall protein content is often reduced (Vierling, 1991; Gulen and Eris, 2004; Kotak et al., 2007). Here, in contrast, we found that total protein content is strongly influenced by temperature, exhibiting a positive correlation and reaching its highest level at 55°C, significantly higher than Control levels (**Figures 7**, **8B**; **Table 1**). This pattern may reflect the accumulation of stress-related proteins. However, it is important to emphasize that the present study quantified total nitrogen-based protein without distinguishing between functional storage proteins and stress-induced proteins. Therefore, although quinoa leaves maintained high protein levels following severe heat stress, the nutritional quality and digestibility of these proteins remain to be determined.

The findings of this study have important agronomic implications. With the projected increase in the frequency and severity of heatwaves under climate change (Jagadish et al., 2020; Grüter et al., 2022), the ability of YVQ to withstand acute heat stress with no visible damage and while retaining high protein levels underscores its potential as a climate-resilient leafy crop. Since YVQ has a short growth cycle (typically harvested 30–62 DAS, depending on the growing season) (Rubinovich et al., 2023, 2025), its resilience to transient stress events makes it particularly suitable for Mediterranean and arid regions, where extreme heat events often coincide with key growth periods. Moreover, the retention of high protein concentrations even at extreme temperatures suggests that nutritional quality may be preserved, supporting its role as a sustainable alternative to conventional leafy greens. While this study provides important insights, experiments were conducted under controlled growth conditions and assessed short-term responses to heat exposure (5 days), which cannot fully replicate the complexity of field environments where heat stress often coincides with drought, high irradiance, or nutrient limitations (Cohen et al., 2021; Zandalinas and Mittler, 2022). Moreover, quinoa is known to exhibit substantial genotype-specific variation in heat tolerance (Eustis et al., 2020; Matías et al., 2021b). Therefore, the present findings should not be generalized to all quinoa genotypes. Future work should compare quinoa genotypes to identify genetic variation in heat tolerance.

## 5 Conclusion

Overall, these findings support the potential of YVQ as a novel, climate-resilient leafy crop, particularly in regions that are susceptible to increasing heat extremes. In contrast to grain-producing quinoa, for which high temperatures during flowering and seed filling have been linked to substantial yield reductions—a major barrier to its global expansion (Fuentes and Bhargava, 2011; Hinojosa et al., 2018, 2019; Alvar-Beltrán et al., 2020; Matías et al., 2021b), YVQ shows promise as a viable crop under warming and extreme climatic conditions. It can maintain nutritional quality under severe heat stress and serves as a promising model for investigating plant resilience to acute thermal events. Moderate heat exposure (40°C) did not appear to induce a meaningful adaptive response, as physiological parameters remained close to control levels with no evidence of acclimation. In contrast, exposure to 43–49°C elicited clear short-term stress responses, followed by partial recovery over time, suggesting the activation of protective and repair mechanisms typical of short-term heat adaptation. However, at the extreme temperatures of 52–55°C, the damage exceeded the plants’ compensatory capacity, resulting in irreversible impairment of carbon assimilation and membrane integrity. Notably, protein content reached its highest values with the 55°C treatment, suggesting the accumulation of stress-related proteins as part of the plant’s terminal response to severe heat stress. Although days with peak temperatures of 55°C are currently rare and confined to specific geographical regions, recent climate models predict an increase in such extreme events in the coming years (Jagadish et al., 2020; Grüter et al., 2022). Therefore, future studies should focus on investigating and assessing the resilience of different quinoa accessions, along with its wild relatives that are tolerant to extreme heat (Curti et al., 2022; Xu et al., 2025), under both controlled and field conditions, to better prepare for the anticipated impacts of global warming. Future work should also incorporate additional quantitative indices, such as chlorophyll extraction and leaf relative water content, to further complement the non-destructive measurements used in this present study.

## Data Availability

The original contributions presented in the study are included in the article/**Supplementary Material**. Further inquiries can be directed to the corresponding author.

## Supplementary material

Supplementary Figure 1 Recorded relative humidity for the different high-temperature gradients in the controlled-climate growth room. Graph shows representative 24-h relative humidity profiles recorded for each high-temperature gradient treatment.
