# Supplementary figures and images for: Physiological responses of young vegetative quinoa (Chenopodium quinoa Willd.) leaves to high temperatures under controlled conditions
Source: Front Plant Sci. 2026 Jan 12;16:1737240. doi: 10.3389/fpls.2025.1737240 (PMC12833441; doi:10.3389/fpls.2025.1737240)

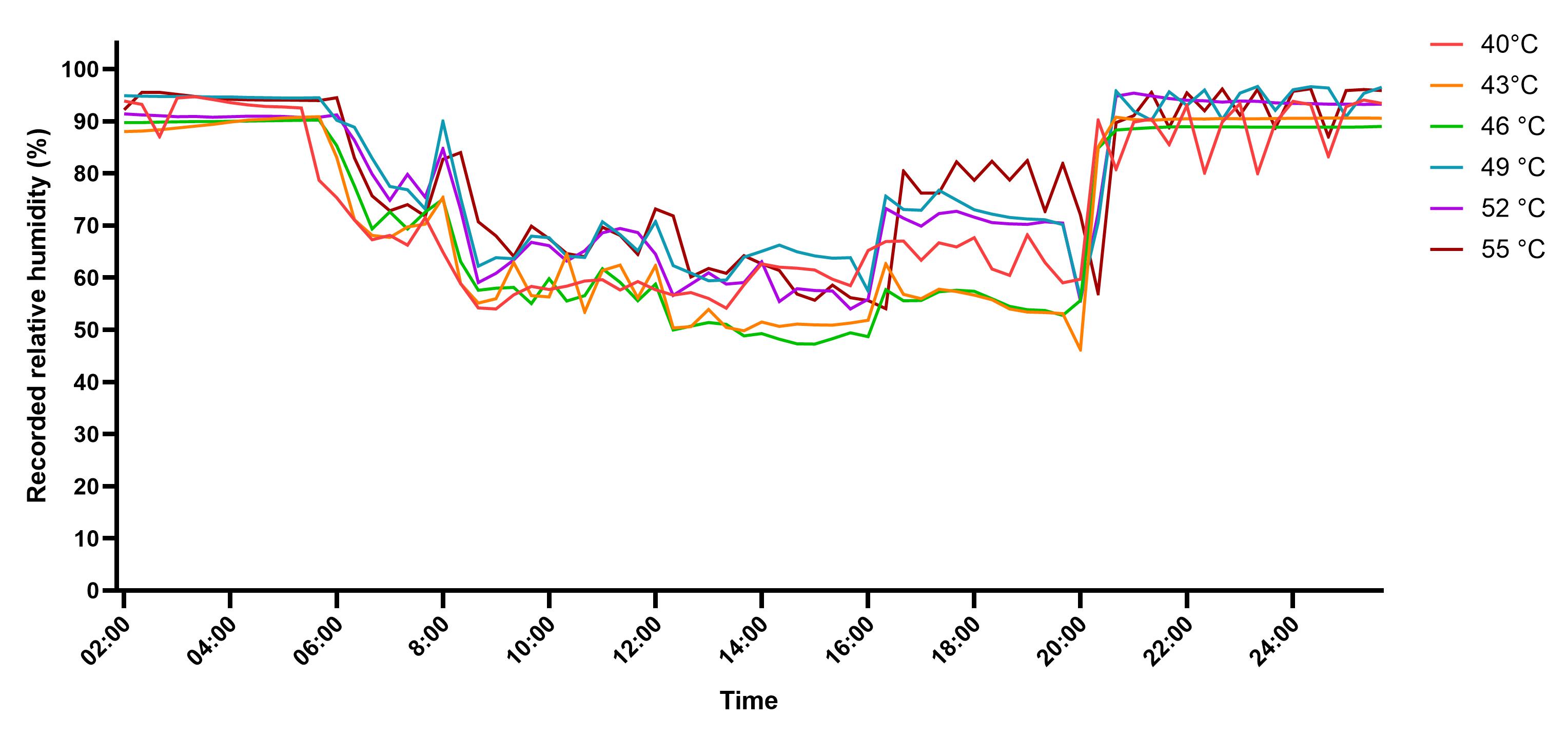

Supplement: Supplementary Figure 1 — Recorded relative humidity for the different high-temperature gradients in the controlled-climate growth room. Graph shows representative 24-h relative humidity profiles recorded for each high-temperature gradient treatment. [file Image1.jpeg]
